# Identifying Priority Areas for the Indian Leopard (*Panthera pardus fusca*) Within a Shared Landscape

**DOI:** 10.1002/ece3.70404

**Published:** 2024-10-10

**Authors:** Aparna Kolekar, Kimberley Hockings, Kristian Metcalfe, Sanjay Gubbi

**Affiliations:** ^1^ Holématthi Nature Foundation Bengaluru India; ^2^ Centre for Ecology and Conservation, College of Life and Environmental Sciences University of Exeter Cornwall UK

**Keywords:** habitat loss, habitat selection, human–carnivore conflict and coexistence, human–leopard interactions, Indian leopard, shared landscape, species distribution modelling

## Abstract

Habitat loss is one of the primary drivers of large felid decline. The leopard (*Panthera pardus*), a generalist large felid species, has the behavioural and dietary flexibility to exploit different habitat types of varying human influence. Understanding habitat selection in a shared landscape is critical for the development of conservation strategies and managing negative human–leopard interactions. The development of conservation policy requires data on large spatial scales, which is mostly lacking, especially within shared landscapes in India. This study aims to determine habitat selection by Indian leopards and the anthropogenic, environmental, and climatic variables contributing to this selection. Leopard occurrence records were obtained from an occupancy survey conducted in the five administrative districts in Karnataka (28,375 km^2^). 267 randomly selected 30 km^2^ grids were each walked for 10 km and all leopard signs were recorded. Environment variables were chosen to reflect land use, climatic, topographic, and human disturbances that could affect habitat selection at a resolution of 0.1 km^2^. The mean ensemble model was projected to the state of Karnataka. Habitat selection predicted by the ensemble model was driven by proximity to forest cover and rocky outcrops, higher precipitation, and negatively by distance to cropland and roads. Protected Areas and Reserved Forests in the study covered 47% of the predicted habitat, while 25% is within human‐use areas such as human habitation and croplands. This study predicts that half of the habitat selected by leopards is outside of protected areas and reserved forests. The selection of human‐use areas is predicted because of the availability of cover from irrigated croplands and the proximity to natural cover that provides refuge. Livestock density did not drive large‐scale habitat selection. The preservation of natural cover and rocky outcrops that provide refuge between protected areas is paramount for leopard conservation.

## Graphical abstract

This manuscript determines the priority landscape for leopard conservation and the factors contributing towards habitat selection. The development of a conservation policy requires data on large spatial scales, which is mostly lacking, especially within India. This study employing fine‐scale data is critical for developing conservation strategies and managing negative human–leopard interactions.

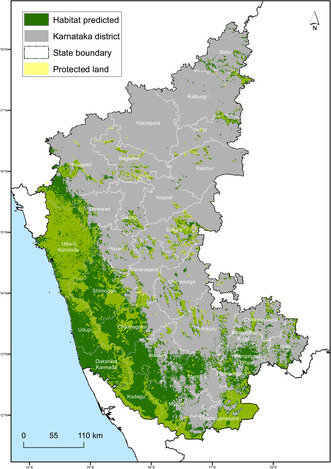

## 1 Introduction

Competition for space is one of the primary factors driving species extinction and negative interactions between large felids and people (Inskip and Zimmermann 2009). The rapid growth of human population and expansion of agriculture and urban areas, mining, and logging have led to the loss of natural habitats for wildlife worldwide (Ceballos and Ehrlich 2002; Morrison et al. 2007). Large felids today occur in depressed numbers largely restricted to reserves or protected areas (PAs), most of which are not sufficiently large or contiguous to hold viable large felid populations (Nowell and Jackson 1996; Ripple et al. 2014; Bauer et al. 2015; and Goodrich et al. 2015). The borders of PAs experience increased negative interactions when large felids, specialised in ungulate predation, switch to killing domesticated livestock due to the ease of predation and increased encounter rate (Treves and Karanth 2003; Inskip and Zimmermann 2009). Habitat degradation further erodes the quality of the animal's habitat. Decreased wild prey abundance can lead to increased livestock predation and even human attacks (Reza, Chowdhury, and Santiapillai 2000; Polisar et al. 2003; Inskip and Zimmermann 2009; Burgas, Amit, and Lopez 2014). The visibility of large carnivores and the potential threat they pose to human lives create a fear psychosis, which can build resentment amongst local communities, making them hostile towards conservation initiatives and PA personnel, inciting retaliatory killings (Madhusudan and Mishra 2003; Woodroffe, Thirgood, and Rabinowitz 2005; Treves et al. 2006; IUCN 2023).

Communities living in the vicinity of PA limits can suffer disruption in their livelihoods due to livestock predation and livestock guarding, fear for their safety, and reduced well‐being. Such negative interactions not only affect the community's sustenance but also reduce the quality of life and mental health, disrupt livelihoods, and cause food insecurity (Barua, Bhagwat, and Jadhav 2013; Kansky and Knight 2014). Communities, especially in poorer countries, lose health and time over guarding livestock and crops (Barua, Bhagwat, and Jadhav 2013). Although felids may not be the most damaging in terms of depredation costs, for example, diseases killed 14% of livestock, while predation by lions accounted for a mere 3% (Woodroffe and Ginsberg 1998), their visibility as large carnivores capable of inflicting threats to human lives creates fear psychosis, and hence they are also the most persecuted (Goldman, De Pinho, and Perry 2013; Kansky and Knight 2014).

The narrative can also be one of tolerance. For example, in India, some local communities have imbibed a degree of tolerance towards large felids through complex cultural beliefs, where hunting large felids is taboo (Bhatia et al. 2013; Ghosal and Kjosavik 2015). However, other regions have reported erosion of such religious taboos (Madhusudan and Karanth 2002). In many cases, lethal control of felids could be limited by people's ability to capture and kill (Woodroffe, Thirgood, and Rabinowitz 2005).

Leopard (*Panthera pardus*), a large felid, can subsist on a wide range of diet and habitat types (Sunquist and Sunquist 1989) and even occur in high‐density human areas such as on the edge of cities (Athreya et al. 2015; Athreya et al. 2013; Gubbi, Kolekar, and Kumara 2020; Kuhn 2014). In India, leopards's ability to tolerate disturbance also allows them to survive alongside people in agricultural areas subsisting on livestock, stray dogs, cats, and pigs and a variety of smaller prey, including field mice (Athreya et al. 2013; Edgaonkar and Chellam 2002; Kshettry, Vaidyanathan, and Athreya 2017; Nowell and Jackson 1996). The pervasiveness of the species, however, has led to a severe overestimation of its status and range (Jacobson et al. 2016). Leopard populations have seen over a 60% decline in the last few decades, largely driven by habitat loss, losing nearly 75% of their former range (Gubbi et al. 2014; Jacobson et al. 2016; Grooten and Almond 2018). Leopard populations are also severely threatened by poaching, which is driven by a market for skins, claws, and bones (Raza et al. 2012; Mondol et al. 2015).

Research on leopard habitat use has primarily focused on PAs or at the borders of PAs (Mondal et al. 2022; Ghosal et al. 2013; Karanth et al. 2013). However, increasing interactions between people and leopards in human‐use areas have shifted the research focus from a PA‐centric focus to a landscape‐level conservation approach (Athreya et al. 2013; Gubbi et al. 2017; Gubbi, Kolekar, and Kumara 2020; Vijayan and Pati 1999). Understanding the factors influencing where they are likely to occur in the shared landscape by modelling habitat selection can help to determine leopard conservation priority areas (Abade et al. 2018; Gubbi, Sharma, and Kumara 2020). In this study, we use ecological niche modelling (ENM) to determine leopard distribution across a shared landscape within Karnataka, southern India.

Machine learning tools used in ENM can map exotic species invasion and unknown distributions of species (Guisan and Zimmermann 2000; Soley‐Guardia, Alvarado‐Serrano, and Anderson 2024). ENM utilises occurrence data and determines the realised niche occupied, dependent on the environment surrounding the occurrence (Guisan and Zimmermann 2000; Franklin and Miller 2010). The environmental variables, which can also be climatic, determine the range of values the species can tolerate, or geographical factors and human disturbance factors that limit dispersal. Several algorithms have been developed (Elith et al. 2006; Heikkinen et al. 2006), and ensemble forecasting is used towards a consensus approach (Thuiller 2004).

Using niche modelling, this study aims to identify habitat selection by the Indian leopard (*Panthera pardus fusca*) and determine the conditions that encourage selection within human‐use areas. Such information will help to develop conservation and management priorities for the Indian leopard, especially across human‐dominated landscapes.

Leopard usage of space is highly driven by land use and land cover characteristics, with a higher probability of space use when the proportion of natural cover is high (Thatte et al. 2018; Gubbi, Sharma, and Kumara 2020; Mondal et al. 2022). We hypothesise that “the selection of habitat for the leopard hinges on the availability of natural cover even when they survive in human‐dominated landscapes”. Specifically, the objectives of this study are:

- to identify key environmental and anthropogenic factors that determine *P. p. fusca* habitat use;
- to determine land use characteristics of habitat selection within human‐use areas; and
- to identify priority landscapes within a shared landscape for the species survival.

### 1.1 Study Area

**FIGURE 1:**
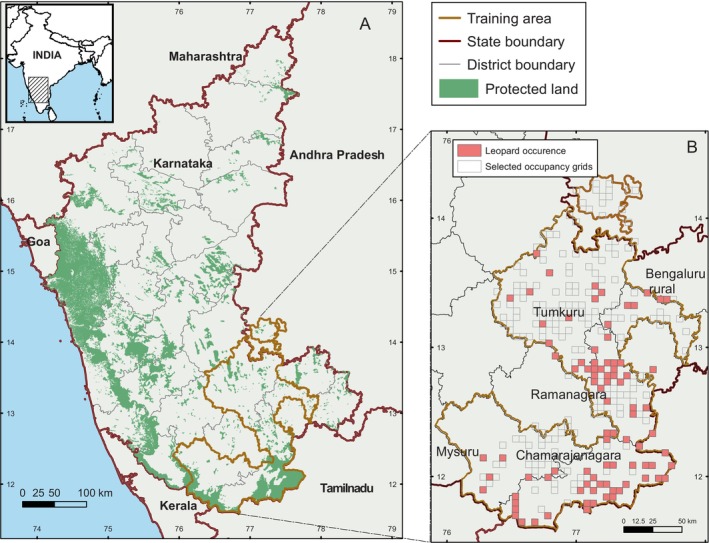
(A) The state of Karnataka, India, showing district boundaries and protected land coverage composed of protected areas, reserves, and state forests. (B) The training area within the study area is composed of the five districts—Tumkuru, Bengaluru Rural, Ramanagara, Mysuru, and Chamarajanagara, shown with occupancy grids that were surveyed. Grids with leopard occurrence are shown in light red.

The study area is the state of Karnataka, India, where leopards occupy nearly half of the state's geographical area, including irrigated croplands, multiple‐use forests, unprotected natural areas, rain‐fed agricultural farmlands, and built‐up areas (Athreya et al. 2015). The state covers an area of 191,791 km^2^ (Figure 1A), flanked by the Western Ghats, a biodiversity hotspot, as well as part of the Eastern Ghats. The Deccan plateau in the state's central region supports meadow grassland habitat and dry thorny scrubland. A large part of the state is cultivated farmlands with a mix of irrigated and rainfed croplands. While PAs cover 5.6%, a combination of PAs and reserved and state forests, hereafter called protected land (PL), covers 11.92% of the geographical area of the state. The state has three climatic classes: arid, semi‐arid, and humid, depending on the amount of rainfall it receives, pre‐monsoon rain (January–May), monsoon rain (June to September)–accounting for the majority of the rainfall from the southwest monsoon winds, and post‐monsoon rain (October to December) from northeast monsoon winds. The mean annual rainfall varies from 400 mm in the Eastern region to more than 4000 mm in the Western Ghats (KSDA 2019).

For training data, we selected five districts from the state—Bangalore rural, Tumkuru, Ramanagara, Chamarajanagara, and Mysuru, wherein the leopard occurrence data were available. The districts cover an area of 28,375 km^2^ (Figure 1B), henceforth referred to as the training area. The training area has a mix of all vegetation types, ranging from grassland with rocky outcrops, dry and thorny scrubland interspersed with agricultural landscape growing rainfed and irrigated crops and plantations, to deciduous and semi‐evergreen forest stretches, representative of the study area. The final results from the training area will be projected onto the study area.

## 2 Methods

Leopard occurrence data were derived from an occupancy field survey conducted between the years 2014 and 2015 (Gubbi, Sharma, and Kumara 2020) within the 28,375 km^2^ training area. The survey divided the training area into 30 km^2^ grids (*n* = 1058). Grids were categorised as predominantly rainfed croplands, irrigated croplands, natural areas (e.g., forests, grasslands, and rocky outcrops) and anthropogenic (containing built‐up areas), using the land use classification. These grids were randomly selected (*n* = 267) with the criteria that all types of the category were equally covered. In all the grids, line transects were conducted on foot, during which observers recorded the presence or absence of indirect signs of the leopard and its prey (e.g., wild prey such as spotted deer (*Axis axis*), sambar deer (*Rusa unicolor*), porcupine (*Hystrix indica*) etc.) and domesticated livestock such as dogs, cattle, sheep, and goats at every 100 m interval. The total distance walked within each grid was 10 km. The distance walked within each grid in each category was dictated by the percentage of the category in the grid. For example, if grid 1 contained 30% irrigated croplands, 40% rainfed croplands, and 30% natural area, the 10 km transects were walked in 3 km each of irrigated cropland and natural habitat and 4 km within the rainfed croplands. The transect lines were walked along trails and tracks (not roads) to maximise observations (Karanth et al. 2011).

All leopard signs were recorded within a 100 m line segment; hence, we assigned the midpoint of each segment as leopard occurrence (*n* = 512). The occurrence data were significantly clustered (*p* < 0.05). Using the spatial thinning process from the spThin package (Aiello‐Lammens et al. 2015) applied at increments of 100 m, the data set was thinned iteratively at 1 km, resulting in (*n* = 160) presence records. For absence data, the midpoint of all line transects walked in the occupancy survey that did not record leopard signs was extracted. The absence locations too were subsampled by thinning at 1 km. From this, an equal number of records were randomly selected to match the number of the occurrence records. The final presence‐absence data set was tested for spatial autocorrelation using Moran's I.

To realistically reflect the ecology of a generalist species, variables from both climatic and remote sensing data depicting land use must be used, especially if the species is occupying a spatially fragmented habitat (Cord and Rödder 2011). Since the leopard occurrence data from the occupancy survey pertained to the year 2014–15, environmental data were selected that covered this period wherever possible. Selected variables were from land use, climatic, topographic, and human disturbances (Appendix S1).

Topographic features (1) altitude and (2) slope were obtained from the Shuttle Radar Topographic Mission digital elevation model (Farr et al. 2007). Climatic data layers (3) annual precipitation, obtained from CHIRPS Pentad (Huntington et al. 2017); (4) vegetation fraction cover obtained from NRSC (2014), corresponding to the driest month, as a proxy for cover available throughout the year (Filipponi et al. 2018) were used. From the 19 bioclimatic variables (Hijmans et al. 2005), the following variables were selected by determining variable importance using principal component analysis (PCA) (Figure S1); (5) Bio 1: annual mean temperature; (6) Bio 5: maximum temperature of warmest quarter; (6) Bio 7: temperature annual range; (7) Bio 9: mean temperature of the driest quarter; (8) Bio 10: mean temperature of the warmest quarter; (9) Bio 11: mean temperature of coldest quarter; and (10) Bio 18: precipitation of the warmest quarter.

A land use map was downloaded from the land‐use land cover (LULC) for the year 2014–15 provided by the National Remote Sensing Centre (NRSC 2014), from which the following land use categories based on distance were used: (11) distance to plantation; (12) distance to double–triple crop; (13) distance from single crop composed of Kharif, Rabi, Zaid croplands, and fallow; (14) distance to rocky outcrop only– derived from the wasteland category by extracting areas with a slope greater than two degrees; (15) distance to natural areas created by combining—evergreen forests, deciduous forests, scrub forests, and grasslands; and (16) distance from water bodies by combining max and min.

Human disturbance variables used in the model are (17) distance from paved roads excluding track and trails (OpenStreetMap contributors 2019); (18) human density (Census of India, 2011) and livestock density from the 19th livestock census data, 2012; (19) dogs; (20) cattle and buffaloes; (21) sheep and goat; and (22) poultry. Census numbers available at the village level were mapped to the village layer, and density per square kilometre was calculated. The classification of livestock was followed to identify the influence of livestock type and size class on habitat selection by leopards (Hayward et al. 2006; Athreya et al. 2013). Poultry density was used since leopards frequent poultry farms or dump yards closer to such farms (Gubbi et al. 2017) (personal observation ‐ Kolekar, A.).

All environmental data layers were resampled using bilinear interpolation to 100 m due to the uncertainty in the presence of data to an extent of 100 m. The grid resolution is considerably fine for the target species and is a good compromise between accuracy and the time consumed by the modelling process.

A large set of variables leads to an increasingly complex model and model overfitting (Merow et al. 2014). Variable selection by eliminating highly correlated and non‐significant variables was performed using PCA to determine the important variables and variance inflation factors (VIFs) using a vifcor |*r*| > 0.7 and vifstep (th = 3) function to eliminate highly correlated variables (Naimi et al. 2014). The variable importance test provided in the biomod2 package (Thuiller et al. 2016) in the R platform (R Core Team 2018) was run on each model iteration by fitting all uncorrelated variables obtained from vifstep to eliminate lower quality variables that were not significant to the model habitat selection by the leopard.

Highly correlated variables were modelled separately to determine their importance. The density of humans, dogs, cattle, and buffalo, precipitation and temperature variables, and distance from built‐up areas and paved roads were highly correlated. Each pair of correlated variables was modelled individually to determine their contribution to the model and, hence, their importance. Based on variable importance, precipitation, precipitation of the warmest quarter, mean temperature of the coldest quarter, vegetation fraction cover, and distances to double–triple crop, forests, plantations, rocky outcrops, and roads were retained to perform model calibration and projection (Figure S2). Variable response was tested by keeping the rest of variables constant following the evaluation strip method (Elith et al. 2005).

### 2.1 Model Selection and Prediction

The modelling approach followed for this study is detailed by Guisan et al. (2017). The Biomod2 package was used since the package provides an inbuilt suite of ENM algorithms and a platform for the ensemble and evaluation of the projections (Thuiller et al. 2016). All algorithms from the package were used during the initial run, later retaining only those algorithms that produced a Test Skill Statistic score (TSS) > 0.7, which were the generalised linear model (GLM), generalised additive model (GAM) and boosted regression tree (BRT), flexible discriminant analysis (FDA), random forests (RF), and multivariate adaptive regression splines (MARS). The model runs were repeated 10 times to obtain a mean estimate of the predictive performance of the model and an assessment of the sensitivity of the model to the occurrence data. From the selected algorithms, only the top models with TSS > 0.7, were used for the final ensemble (for model score, see Appendix S2). The model ensemble used the total consensus or mean of all the selected models (Thuiller et al. 2016).

Prediction accuracy for the consensus model was estimated by determining the sensitivity and specificity of the model and by plotting the area under the receiver operating curve (ROC) to obtain the are under the curve (AUC) score. The evaluation sets used were—a test set comprising 25% of the occurrence data set aside before model calibration and an independent set‐ that was not previously used to calibrate the model. The independent set is a subset of leopard occurrence points from a camera trap exercise conducted in the same year in the training area (Gubbi et al. 2017) and leopard capture locations obtained from government records across the state (Gubbi, Kolekar, and Kumara 2020). The prediction accuracy on the independent set was estimated by determining the sensitivity and specificity of the model from the biomod2 package using a confusion matrix by setting a probability threshold. The final ensemble was projected on the study area using the mean of probabilities over the selected models.

### 2.2 Land Use under the Predicted Habitat

Using zonal statistics and the tabulate area tool in ArcGIS 10.1, the area under the land use category from the predicted habitat was determined for the study area, districts, and PLs. The priority area for leopard conservation was determined by identifying the largest contiguous habitat outside of PLs predicted by the model for natural areas only by combining forests and wasteland.

## 3 Results

### 3.1 Variable Contribution and Response

**FIGURE 2:**
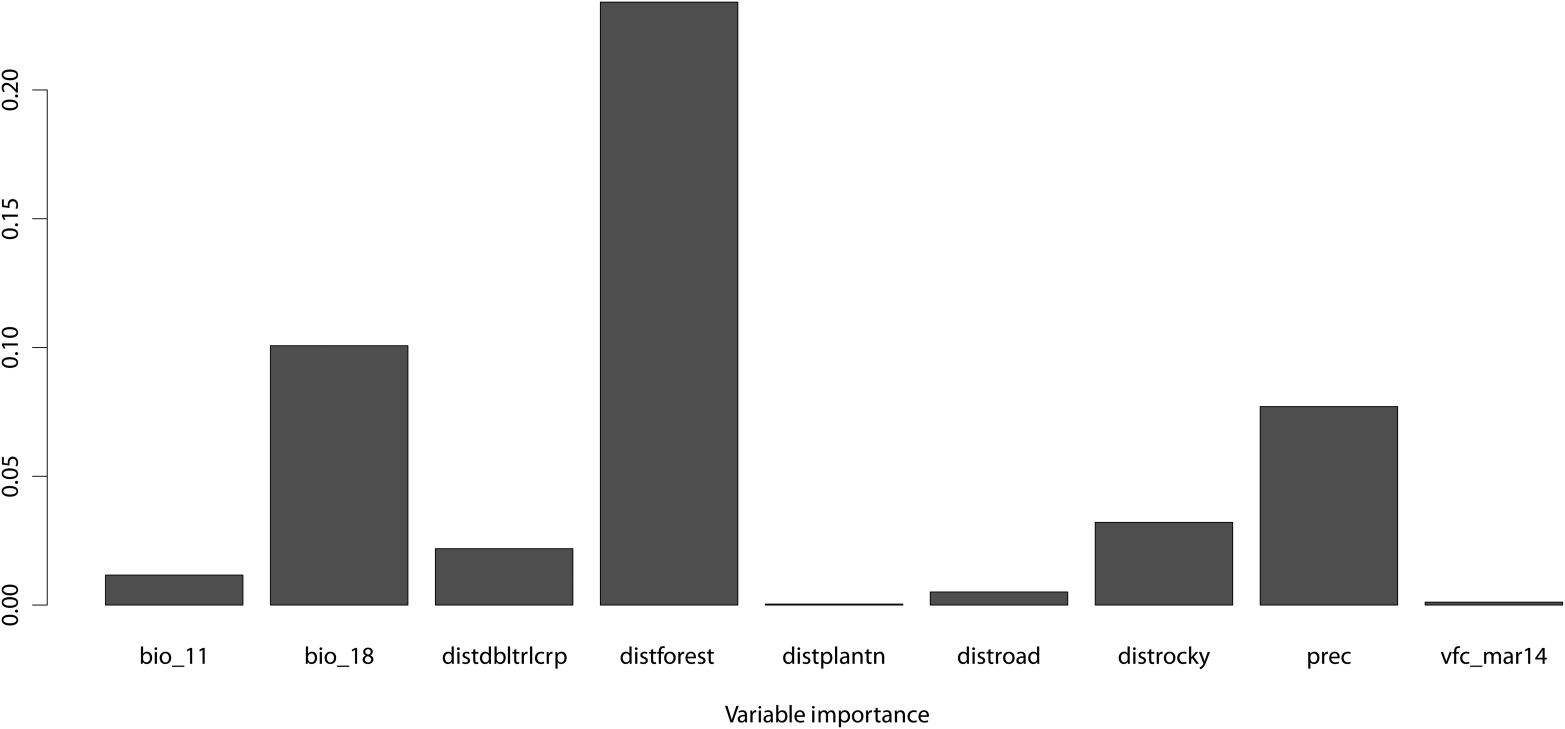
Variable importance with percent contribution for the consensus model to determine habitat selection by *Panthera pardus fusca*. Variables—bio_11: mean temperature of the coldest quarter, bio_18: precipitation of the warmest quarter, distdbltrlcrp: distance to double–triple crop, distforest: distance to the forest, Distplantn: distance to plantation, distrocky: distance to rocky outcrops, prec: annual precipitation, vfc_mar14: vegetation fraction cover from March 2014.

Amongst the variables selected for the ensemble, distance from forested areas and precipitation of the warmest quarter contributed to 69.16% of the model (Figure 2). The other variables that contributed > 5% were distance to rocky outcrops, distance to double–triple crops, and annual precipitation. Variable contributions from all selected variables are provided in the Supporting Information (Appendix S3).

**FIGURE 3:**
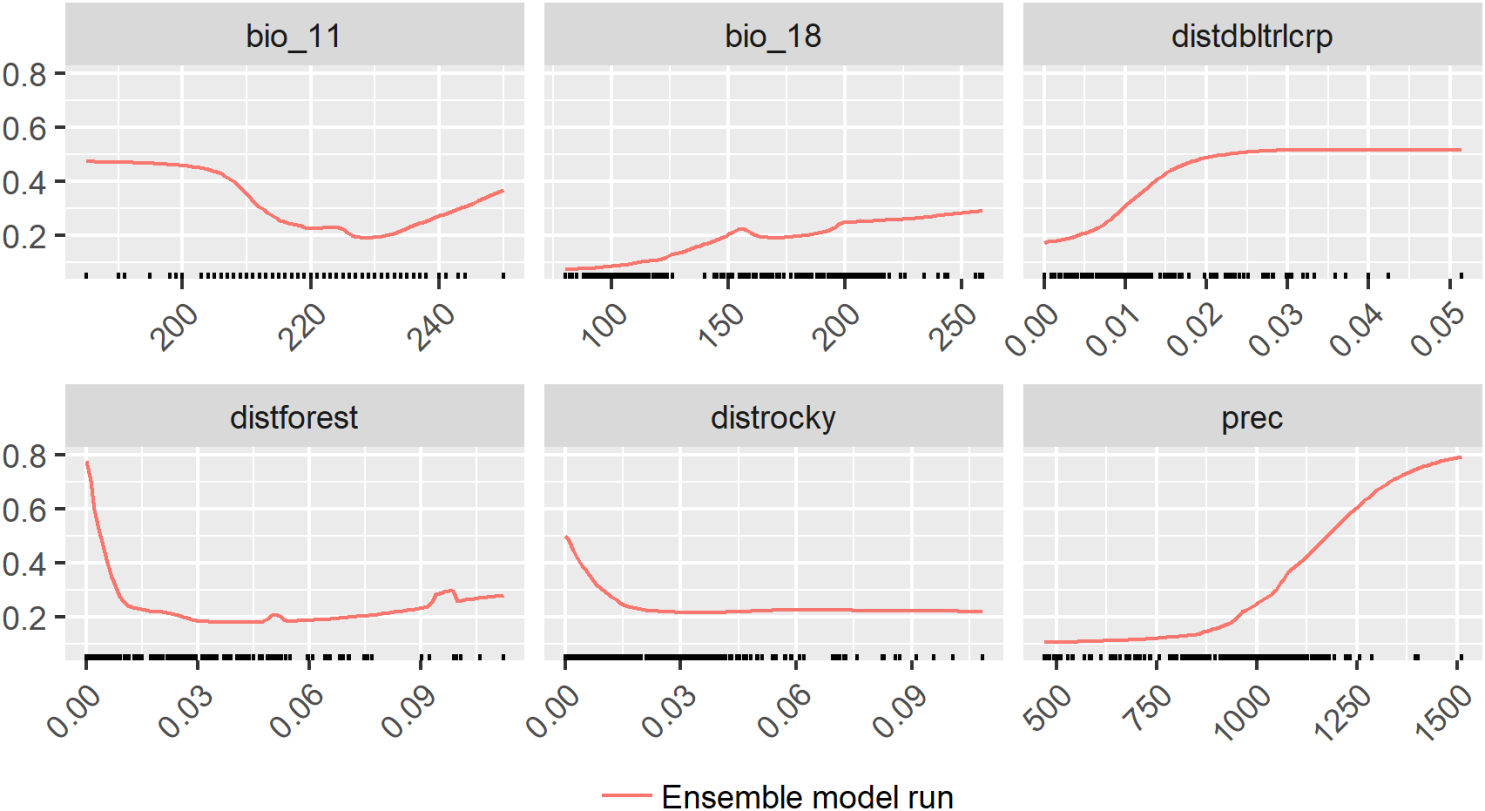
Variable response from the consensus model shows the response for each of the variables that contributed significantly (% contribution > 4, see Appendix S2) when all other variables are held constant (Elith et al. 2005). Variables—distdbltrlcrp: distance to double–triple crop, distforest: distance to the forest and distrocky: Distance to rocky outcrops. The distance on the X axis is in WGS decimal degrees, where 0.01 = 1.06 km. prec: annual precipitation and bio_18: precipitation of the warmest quarter. The ‘X’ axis unit is in millimetres. bio_11: mean temperature of the coldest quarter.

Variable response shows habitat selection by the leopard is significantly negatively influenced by distance to the natural cover provided by forested areas and positively associated with higher annual precipitation (Figure 3). The effect of distance from double–triple cropland (when the rest of the variables are constant) is no longer a factor after a distance of 2 km or more reaching asymptote. The predicted habitat quality is higher in regions that are forested and receive annual rainfall of 800 mm or above. The response of variables whose contribution is < 5% varies with different algorithms; GLM, FDA, and GAM predict that double–triple croplands provide a lower quality habitat, with the quality increasing at > 1 km distance; GLM and GAM predict that the quality of habitat decreases with increasing distance from rocky outcrops. For variable response from each of the selected algorithms (Figure S3).

### 3.2 Ensemble Model Projection

The predictive performance of the ensemble tested using the test set showed 95.87% sensitivity and 89.69% specificity against a mean TSS. On testing the model against the independent test set, the model showed 79.3% sensitivity and 89% specificity. The confusion matrix on the independent test set evaluated the model and predicted absence for a true positive 11% of the time and false presence 20% of the time. The ROC curve gave an AUC score above 0.91, indicating high accuracy (Figure S4). To generate a binary prediction model, the mean probability cutoff of 0.47 predicted from the independent evaluation test was used.

**FIGURE 4:**
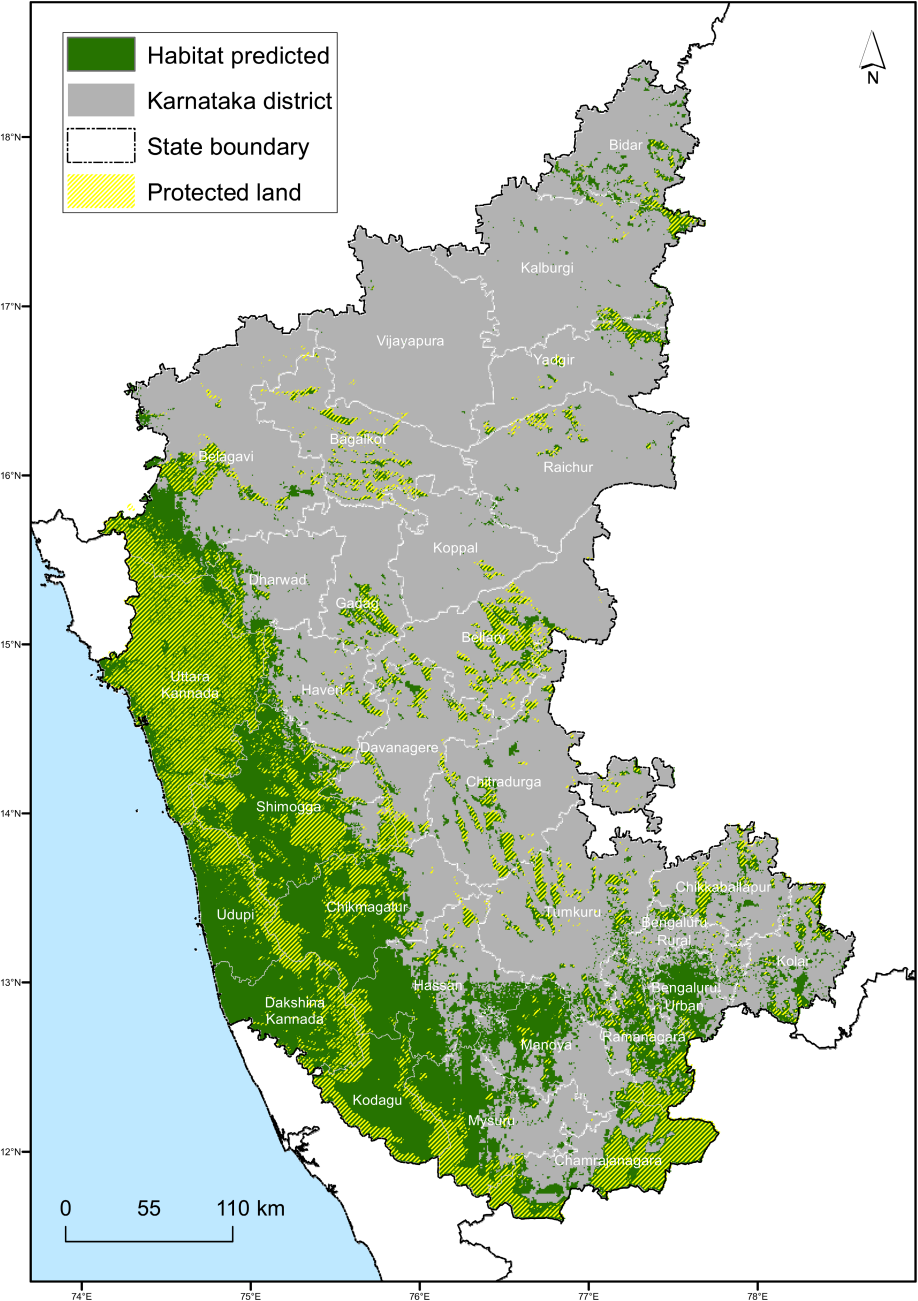
Binary projection from the mean ensemble of models on the study area shown in green with protected land comprising of protected area, reserves, and state forests shown in hatched yellow lines and district boundaries.

Using the threshold determined by the mean probability cutoff from the evaluation of the independent test set, the habitat projected for the study area covers 29.72% of the state of Karnataka (Figure 4). Forest habitat contributed 61.69% of the predicted habitat, while croplands, not including plantations, contributed 21.84%. Within the wasteland category, 76.66% of the predicted habitat is a rocky outcrop, and the rest is degraded pasture. The grassland and waterbodies min category were omitted by the model from habitat selection. For model projection using a continuous habitat quality index, see Figure S5.

A comparison between the PL landscape in the study area and the predicted habitat shows that 83.8% of PL is a suitable habitat for leopards. An area of size 30,495 km^2^ (53.80% of total suitable habitat) outside the PL is suitable for leopard habitat, out of which 43.95% is natural habitat (forest and wasteland combined). The predicted habitat in human‐use areas (built‐up, plantations, and croplands = 31.75%) is clustered around the natural habitat; the maximum distance at which human‐use habitat is predicted from the natural is 22.3 km (Figure S6). Percent land use within the predicted habitat and percent land use type within habitat outside PL are given in Table 1.

**TABLE 1:** Percent area predicted as habitable from the total land use category in the study area, and percent area predicted as habitable available outside protected land (protected area and reserves and state forests) from the land use categories. Here, single crop is composed of Zaid, Kharif, Rabi, and fallow croplands; forest is composed of evergreen, deciduous, and scrub forests; wasteland comprises pasture land and rocky outcrops; and waterbodies include the waterbody and its flood plain.

Land use category	(%) area habitable within each category in the study area	(%) the habitable area outside protected land
Built‐up	2.17	1.98
Single crop	11.35	8.98
Double–triple cropland	8.80	7.15
Plantation	7.44	6.82
Forest	62.83	23.88
Wasteland	6.21	3.05
Waterbodies	1.19	0.89

Area predicted as suitable for leopard habitat was highest within the districts in the Western Ghats—Udupi (95%), Uttara Kannada (95%), Dakshin Kannada (95%), Kodagu (94%), and Shimogga (83%), and lowest in districts in the Deccan Plateau—Vijayapura (0.001%), Raichur (0.01%), and Yadgir (0.02%), amongst others. A breakdown of per cent land use category within each of the districts is given in the Figure S2 and Appendix S4.

### 3.3 Priority Areas for Leopard Conservation

**FIGURE 5:**
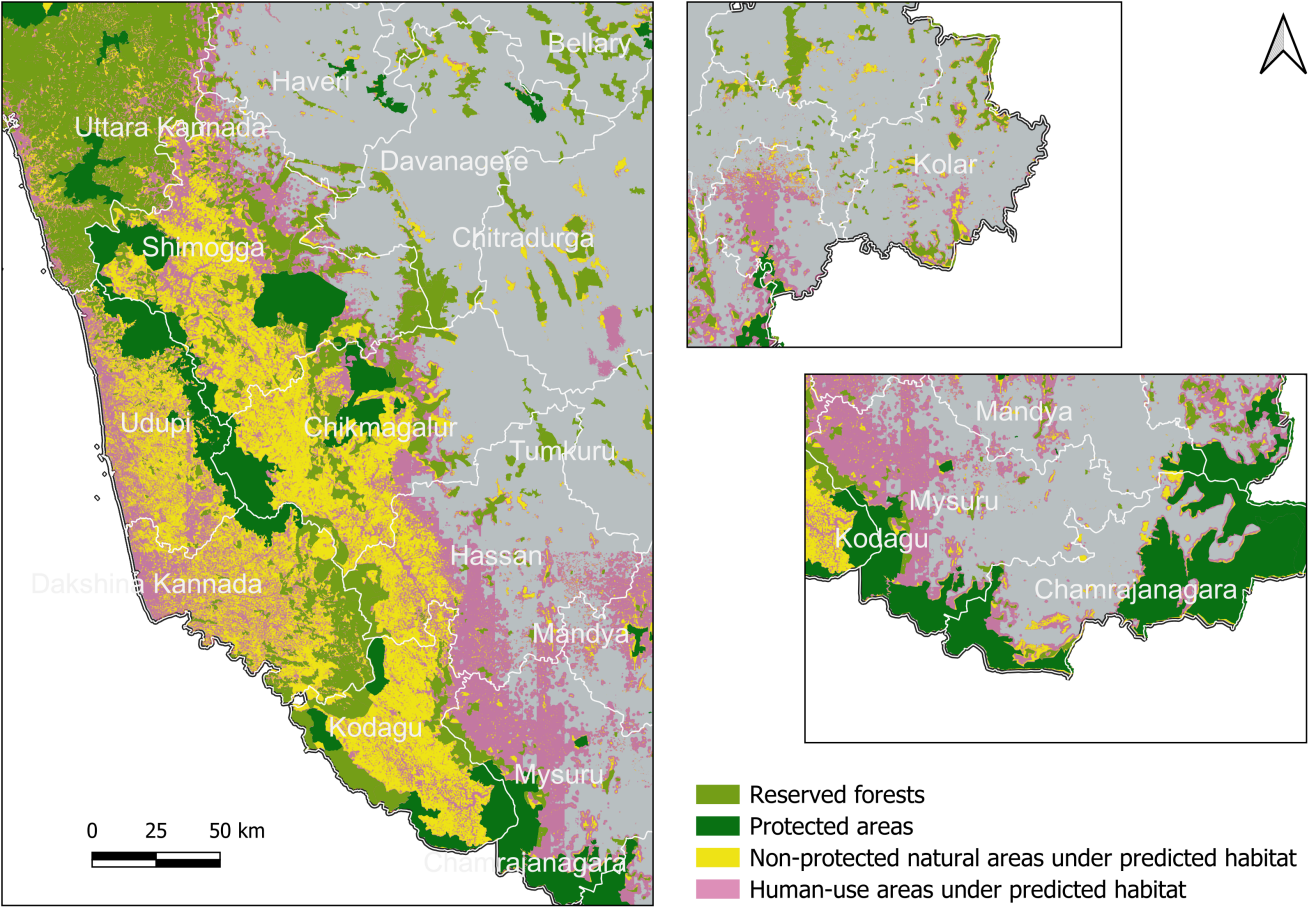
Contiguous leopard habitat with areas > 80 km^2^ outside of protected areas, reserves, and state forest coverage in the study area. Human‐use areas predicted as habitat were discarded, combining only forests, rocky outcrops, and degraded pastures from the mean ensemble model.

On extracting the contiguous habitat available from the modelled habitat using forest and wasteland categories, 818 blocks were identified; five blocks span an area > 50 km^2^, out of which the block within the Western Ghats is the largest contiguous natural, unprotected block spanning 13,042 km^2^ (Figure 5). These could contain private and revenue land holdings.

## 4 Discussion

The results from habitat suitability modelling reinforce the importance of both protected and multiple‐use areas for leopard conservation. Annual rainfall patterns and forests were strongly associated with leopard distribution within the study area. Leopard habitat selection was driven by areas with annual precipitation above 800 mm and with a high precipitation rate during the warmest quarter that roughly correlates with the summer months from January to April. Higher precipitation during the warmer months could support flora and provide cover for leopards, perhaps driving the selection of this bioclimatic indicator for the model. It is also possible that the survey conducted during the dry seasons between March–June 2014 and September–November 2014 to maximise the chances of detecting signs could have played a role in increasing the chances of seasonal habitat selection in the model towards areas that see pre‐ and post‐monsoon showers.

Rocky outcrops classified under wasteland accounted for 3.83% of the suitable habitat. This habitat type is under immense pressure from the granite mining industry. Mining activities cause disturbance and displacement of wildlife, increasing negative leopard–human interactions in the surrounding region.

Non‐selection of human density and distance from built‐up areas as a factor for habitat selection indicates that leopards can persist along human‐use areas (Edgaonkar 2008; Chapron et al. 2014; Athreya et al. 2015). However, the selection of this land use category was negatively dependent on distance from forests and paved roads. In the Bengaluru Urban district, the habitat selection was mainly from the built‐up category. This region is interspersed with pockets of PL, natural areas, and rocky outcrops, perhaps encouraging habitat selection within this anthropogenic landscape.

While leopards are known to primarily subsist on domestic livestock and dogs in a heavily human‐modified landscape (Athreya, Society, and Belsare 2007; Thapa 2011) by attacking smaller isolated livestock holdings, especially if there is available cover (Kolowski and Holekamp 2006), livestock density in this study did not significantly impact habitat selection by leopards. The ubiquitous presence of livestock and domestic dogs throughout the study area perhaps lowered the influence of livestock density.

This study emphasises that shared landscapes are vital for the survival of the species. A quarter of the habitat predicted as suitable for leopards is within plantations and croplands. However, the selection of croplands is dependent on the proximity to forested patches and rocky outcrops, suggesting (natural) permanent cover is vital for leopard's habitat selection within this shared landscape. Proximity to the natural cover provided by forests and rocky outcrops has allowed leopards to occupy croplands such as the double–triple crop category, perhaps seasonally, whenever the vegetative cover is available for an extended period. Double–triple crop category land is croplands that were recorded with crops all year round. The major crops grown in such farmlands in the study area are paddy, tur, ragi, maize, sugarcane, fodder, green gram, horse gram, etc., (KSDA 2019). Crops such as green gram, groundnut, paddy, etc. do not provide enough cover for leopards. Sugarcane fields and maize croplands have, on the other hand, reported breeding leopard populations since they provide a modicum of cover with large periods of decreased human activity (Athreya et al. 2013, 2016; Kshettry, Vaidyanathan, and Athreya 2017). However, since even such irrigated crops are harvested, the resulting loss of habitat can expose the animal, leading to increased negative interactions and calling for animal capture. The brunt of conflict and conservation in such cases are disproportionately suffered by poor marginal communities. Annually, an average of 46 leopards were captured from human‐use areas and translocated; 6.5% of these cases were a result of human injury and death in Karnataka (Gubbi, Kolekar, and Kumara 2020). Erosion of tolerance is perhaps the leading cause of retaliatory killings by communities (6% of leopard deaths annually) in Karnataka (Athreya et al. 2015).

A previous study that used media reports to determine the occupancy of leopards at the level of sub‐districts within Karnataka predicted 47% leopard occupancy within the state (Athreya et al. 2015). However, using media reports has its own limitations as it does not consider variables such as ecological features, climatic conditions, reporting centres, etc., hence may not represent a true picture. This study builds on these findings and employs fine‐scale data and land use categories. This study identified 30% of the state as a suitable leopard habitat for occupancy. Even with a liberal cutoff threshold provided by the model evaluation using the test set, the percentage of suitable habitat for occupancy is 33.5%. Hence, considering fine‐scale ecological data is critical for species distribution studies as it can have conservation management implications.

Though the data provided in this manuscript is about a decade old, the study results act as a benchmark against which future comparisons of leopard occupancy can be made. Such comparative studies will be invaluable in a fast‐changing society and economy like India.

The results from this study highlight that despite being branded as a habitat generalist with high tolerance towards human disturbance, the leopard preferentially selects natural cover over croplands. However, leopards can select suboptimal habitats in the proximity of natural cover. Habitat suitability can positively affect leopard occupancy and, hence, interactions with people. Identification of contiguous natural areas outside of PLs from this study can aid in targeted management to protect vital contiguous habitats outside of PLs that will promote species survival and prioritise limited conservation resource allocation to achieve targeted conservation interventions.

## Author Contributions

**Aparna Kolekar:** conceptualization (lead), data curation (lead), formal analysis (lead), funding acquisition (lead), investigation (lead), methodology (equal), project administration (equal), resources (lead), software (lead), validation (lead), visualization (lead), writing – original draft (lead), writing – review and editing (lead). **Kimberley Hockings:** conceptualization (equal), project administration (equal), resources (supporting), supervision (lead), validation (equal), visualization (lead), writing – original draft (supporting), writing – review and editing (equal). **Kristian Metcalfe:** conceptualization (equal), formal analysis (supporting), methodology (lead), project administration (equal), supervision (equal), validation (equal), visualization (equal), writing – original draft (supporting), writing – review and editing (supporting). **Sanjay Gubbi:** conceptualization (supporting), project administration (supporting), resources (equal), supervision (equal), validation (supporting), visualization (supporting), writing – original draft (supporting), writing – review and editing (equal).

## Conflicts of Interest

The authors declare no conflicts of interest.

## Supporting information


Appendix S1.

Figure S1.

## Data Availability

All data and R code used in the analyses have been uploaded to Dryad data repository and private for peer review at https://datadryad.org/stash/share/H68FLm9SvXVh3uKcgM9vo5satXcQYXMHJL0CvGNTND0. Reference number: doi:10.5061/dryad.80gb5mkxv.
