# Correction to Identifying Priority Areas for the Indian Leopard (*Panthera pardus fusca*) Within a Shared Landscape

**DOI:** 10.1002/ece3.70579

**Published:** 2024-11-21

Kolekar, A., Hockings, K., Metcalfe, K. and Gubbi, S. (2024), Identifying Priority Areas for the Indian Leopard (*Panthera pardus fusca*) Within a Shared Landscape. Ecol Evol, 14: e70404. https://doi.org/10.1002/ece3.70404.

Sanjay Gubbi has been incorrectly affiliated. The correct affiliation is Holématthi Nature Foundation, Bengaluru, India.

Under funding, it has been incorrectly stated that the authors received no specific funding for this work. The leopard presence and absence data for this study were collected through the funding provided by Whitley Fund for Nature.

We apologize for this error.

