# Supplementary material for: Identifying Priority Areas for the Indian Leopard (Panthera pardus fusca) Within a Shared Landscape
Source: Ecol Evol. 2024 Oct 10;14(10):e70404. doi: 10.1002/ece3.70404 (PMC11467164; doi:10.1002/ece3.70404)
Supplement: Supplementary file 1 — Appendix S1. [file ECE3-14-e70404-s002.docx]

## **Appendix S1**: Description of environmental covariates considered and the process followed to create the variables and source.

| **Variable name** | **Variable description** | **Variable creation process** | **Source** |
| --- | --- | --- | --- |
| Altitude | Altitude in meters | Resampled | SRTM (30m) |
| Slope | The slope layer was created using the ArcGIS slope tool from the altitude layer. | Resampled | SRTM (30m) |
| Precipitation (prec) | The dataset combines satellite imagery with in-situ station data to provide total rainfall in mm | Resampled | CHIRPS Pentad, Climate Engine, 2014 (4800m) |
| Vegetation Fraction Cover (vfc_mar14) | VFC can be used to reliably determine changes in vegetative cover. The variable was derived from the data corresponding to March 2014. | Resampled | NRSC 2014 (250m) |
| bio_1 | Annual Mean Temperature |  | WorldClim current data, (30 arc seconds) |
| bio_5 | Max Temperature of Warmest Month | Resampled | WorldClim current data, (30 arc seconds) |
| bio_7 | Temperature Annual Range (BIO5-BIO6) | Resampled | WorldClim current data, (30 arc seconds) |
| bio_9 | Mean Temperature of Driest Quarter, | Resampled | WorldClim current data, (30 arc seconds) |
| bio_10 | Mean Temperature of Warmest Quarter | Resampled | WorldClim current data, (30 arc seconds) |
| bio_11 | Mean Temperature of Coldest Quarter | Resampled | WorldClim current data, (30 arc seconds) |
| bio_18 | Precipitation of Warmest Quarter | Resampled | WorldClim current data, (30 arc seconds) |
| Plantation (distplantn) | Monoculture plantations and mixed timber plantations. | Euclidean distance in WGS84 | NRSC 2014-15 (30m) |
| Double triple crop (distdbltrlcrp) | Irrigated farmlands that harvested multiple crops throughout the year | Euclidean distance in WGS84 | NRSC 2014-15 (30m) |
| Single crop (distsinglcrp) | Rain-fed croplands – Rabi, Kharif, Zaid and uncultivated croplands were combined. Kharif (Aug-Nov) and Rabi (Jan-Mar) crops are short-period crops grown during the monsoon and post-monsoon months respectively for 3-4 months. Zaid (Apr-May) crops are irrigated crops that do not depend on the monsoon for water and are generally grown throughout the year between the Rabi and Kharif crops. | Euclidean distance in WGS84 | NRSC 2014-15 (30m) |
| Rocky outcrop (distrocky) | Rocky outcrop was available under the wasteland category lumped with thorny vegetation and degraded pastures. To derive this layer alone pixels that had a slope greater than two degrees were extracted, largely eliminating most of the pasture lands. | Euclidean distance in WGS84 | NRSC 2014-15 (30m) |
| Forest (distforest) | Areas categorized as Evergreen forest, Deciduous forest, Scrub forest and Grassland | Euclidean distance in WGS84 | NRSC 2014-15 (30m) |
| Waterbody (distwater) | Combined minimum and maximum waterbody layers. The layer extracted from LULC shows water bodies that are at least 30m wide only | Euclidean distance in WGS84 | NRSC 2014-15 (30m) |
| Road (distroad) | Paved road layer is created by combining trunk, primary, secondary, tertiary, residential, motorway, service, and link roads. I did not consider tracks and roads labelled as Unknown category. | Euclidean distance in WGS84 | Open Street Map |
| Human population (humandens) | The latest village layer available was from the 2001 census and was mapped with the 2011 population census. The town census data were summed from the ward level and mapped to individual towns. | Density per square kilometre | Office of the Registrar General & Census Commissioner, India, Ministry of Home Affairs, Government of India. Census of India, 2011. Villages / Towns Directory - 2011. http://censusindia.gov.in/2011census/censusdata2k11.aspx |
| Domestic dogs (dogsdens) | Feral and pet dogs | Density per square kilometre | Ministry of Agriculture, Department of Animal Husbandry, Dairying and Fisheries. 19th livestock census data, 2012 |
| Cattle and buffalo (catalodens) | Combined cattle and buffalo including strays | Density per square kilometre | Ministry of Agriculture, Department of Animal Husbandry, Dairying and Fisheries. 19th livestock census data, 2012 |
| Sheep and goats (shoatsdens) | Combined sheep and goat census | Density per square kilometre | Ministry of Agriculture, Department of Animal Husbandry, Dairying and Fisheries. 19th livestock census data, 2012 |
| Poultry (poultrydens) | Combined all poultry data from chicken, turkey, ducks and emu. Household and commercial farms included | Density per square kilometre | Ministry of Agriculture, Department of Animal Husbandry, Dairying and Fisheries. 19th livestock census data, 2012 |

## **Appendix S2:** Model TSS score from the selected top algorithms- Generalised Linear Model (GLM), Generalised Additive Model (GAM) and Boosted Regression Tree (BRT), Flexible Discriminant Analysis (FDA), Random Forests (RF) and Multivariate Adaptive Regression Splines (MARS)Only the models with TSS score > 0.7 were selected for ensemble.

| Algorithm | **RUN1** | **RUN2** | **RUN3** | **RUN4** | **RUN5** | **RUN6** | **RUN7** | **RUN8** | **RUN9** | **RUN10** |
| --- | --- | --- | --- | --- | --- | --- | --- | --- | --- | --- |
| GLM | 0.639 | 0.669 | 0.611 | 0.683 | 0.661 | 0.597 | 0.764 | 0.55 | 0.581 | 0.742 |
| GAM | 0.614 | 0.672 | 0.633 | 0.731 | 0.714 | 0.6 | 0.631 | 0.731 | 0.6 | 0.767 |
| GBM | 0.686 | 0.689 | 0.681 | 0.589 | 0.736 | 0.658 | 0.736 | 0.683 | 0.628 | 0.764 |
| FDA | 0.689 | 0.747 | 0.631 | 0.647 | 0.794 | 0.611 | 0.789 | 0.786 | 0.742 | 0.794 |
| RF | 0.714 | 0.733 | 0.608 | 0.644 | 0.744 | 0.683 | 0.767 | 0.681 | 0.661 | 0.664 |
| MARS | 0.661 | 0.742 | 0.714 | 0.675 | 0.844 | 0.606 | 0.789 | 0.658 | 0.689 | 0.761 |

**Appendix S3**: Variable contribution to the mean ensemble model

| **Variable** | **Mean** | **Percentage contribution** |
| --- | --- | --- |
| Distance to forest | 0.23 | 48.36 |
| Precipitation of the warmest quarter | 0.10 | 20.80 |
| Annual precipitation | 0.08 | 15.92 |
| Distance to rocky outcrops | 0.03 | 6.63 |
| Distance to double-triple croplands | 0.02 | 4.52 |
| Mean temperature of coldest quarter | 0.01 | 2.41 |
| Distance to paved roads | 0.01 | 1.05 |
| Vegetation fraction cover for March 2014 | 0.00 | 0.23 |
| Distance from plantation | 0.00 | 0.08 |

**Appendix S4:** Percentage area habitable in each of the districts in the study area given by [(Habitat area / Area of district )* 100] predicted by the mean ensemble model for the species Panthera pardus fusca and breakdown of percentage land use category within the habitable area in each of the district. Here, cropland is Rabi, Zaid, Kharif and current fallow croplands combined, natural areas are deciduous, evergreen, grassland and scrub forests combined. The districts are ordered on the largest habitat predicted to the lowest in the state.

| **District** | **% area habitable** | **Built-up** | **Croplands** | **Plantation** | **Wasteland** | **Natural areas** | **Waterbodies max** |
| --- | --- | --- | --- | --- | --- | --- | --- |
| Udupi | 94.81 | 2.35 | 13.76 | 17.49 | 1.13 | 64.42 | 0.84 |
| Uttara Kannada | 94.77 | 1.37 | 13.52 | 1.40 | 0.67 | 81.99 | 1.05 |
| Dakshina Kannada | 94.66 | 4.32 | 5.88 | 20.37 | 1.24 | 67.24 | 0.94 |
| Kodagu | 94.35 | 0.40 | 14.42 | 2.54 | 0.18 | 81.97 | 0.49 |
| Shimogga | 82.81 | 2.12 | 21.07 | 6.99 | 0.44 | 66.24 | 3.13 |
| Chikmagalur | 69.93 | 1.02 | 16.86 | 4.73 | 0.85 | 75.36 | 1.19 |
| Chamrajanagara | 59.97 | 0.23 | 17.10 | 1.32 | 6.81 | 74.11 | 0.43 |
| Hassan | 48.65 | 1.30 | 43.86 | 11.58 | 2.54 | 37.58 | 3.15 |
| Mysuru | 48.05 | 3.32 | 49.34 | 8.36 | 1.71 | 35.33 | 1.94 |
| Ramanagara | 46.86 | 1.20 | 30.03 | 18.58 | 18.08 | 30.64 | 1.47 |
| Bengaluru Urban | 40.67 | 46.83 | 17.48 | 13.22 | 5.84 | 14.40 | 2.22 |
| Mandya | 37.02 | 2.33 | 49.06 | 20.20 | 10.28 | 15.11 | 3.02 |
| Belagavi | 21.42 | 3.24 | 39.05 | 2.39 | 14.65 | 39.60 | 1.08 |
| Kolar | 17.23 | 2.55 | 28.03 | 8.80 | 14.25 | 44.61 | 1.76 |
| Dharwad | 17.06 | 1.86 | 35.43 | 2.67 | 3.11 | 56.13 | 0.80 |
| Haveri | 12.90 | 1.41 | 33.98 | 2.55 | 6.03 | 53.90 | 2.13 |
| Davanagere | 11.48 | 0.32 | 14.26 | 2.51 | 14.96 | 67.62 | 0.33 |
| Chikkaballapur | 9.82 | 0.18 | 10.05 | 1.98 | 37.39 | 49.85 | 0.54 |
| Bellary | 9.53 | 0.22 | 6.67 | 0.02 | 19.76 | 73.26 | 0.08 |
| Gadag | 8.95 | 0.23 | 36.00 | 0.03 | 19.18 | 44.28 | 0.27 |
| Chitradurga | 7.50 | 0.57 | 5.08 | 1.27 | 30.33 | 62.48 | 0.26 |
| Bengaluru Rural | 7.27 | 1.54 | 22.22 | 13.87 | 9.64 | 51.10 | 1.63 |
| Tumkuru | 6.57 | 0.80 | 17.49 | 8.22 | 21.27 | 50.98 | 1.24 |
| Bagalkot | 3.40 | 0.09 | 4.51 | 0.00 | 21.66 | 73.65 | 0.08 |
| Bidar | 1.53 | 0.87 | 58.75 | 2.49 | 22.64 | 14.45 | 0.80 |
| Kalburgi | 0.69 | 2.04 | 29.40 | 0.31 | 3.97 | 61.22 | 3.06 |
| Koppal | 0.31 | 0.00 | 9.76 | 0.00 | 32.25 | 55.96 | 2.03 |
| Yadgir | 0.02 | 0.00 | 27.68 | 0.00 | 14.29 | 58.04 | 0.00 |
| Raichur | 0.01 | 0.97 | 39.81 | 0.00 | 14.56 | 38.83 | 5.83 |
| Vijayapura | 0.00 | 0.00 | 85.71 | 0.00 | 0.00 | 14.29 | 0.00 |
