# Supplementary material for: Identifying Priority Areas for the Indian Leopard (Panthera pardus fusca) Within a Shared Landscape
Source: Ecol Evol. 2024 Oct 10;14(10):e70404. doi: 10.1002/ece3.70404 (PMC11467164; doi:10.1002/ece3.70404)
Supplement: Supplementary file 2 — Figure S1. [file ECE3-14-e70404-s001.pdf]

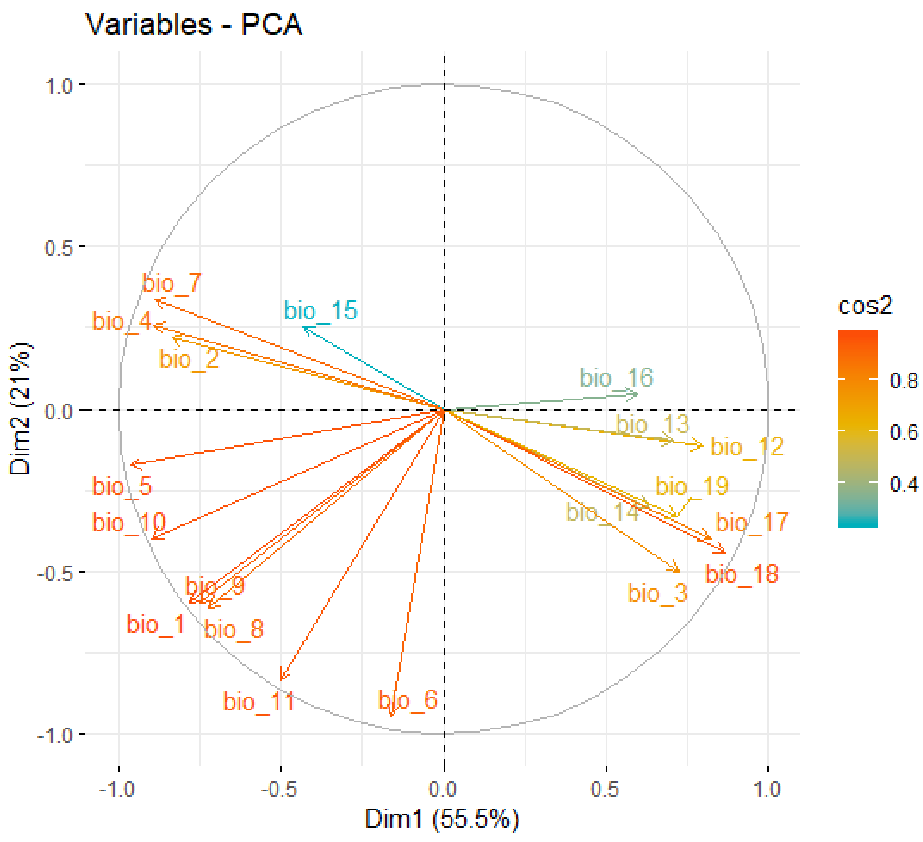

Figure S1: Variable importance using Principal Component Analysis from the 19 Bioclimatic variables

bio_1 = Annual Mean Temperature, bio_2 = Mean Diurnal Range, bio_3 = Isothermality, bio_4 = Temperature Seasonality, bio_5 = Max Temperature of Warmest Month. bio_6 = Min Temperature of Coldest Month, bio_7 = Temperature Annual Range, bio_8 = Mean Temperature of Wettest Quarter, bio_9 = Mean Temperature of Driest Quarter, bio_10 = Mean Temperature of Warmest Quarter, bio_11 = Mean Temperature of Coldest Quarter, bio_12 = Annual Precipitation, bio_13 = Precipitation of Wettest Month, bio_14 = Precipitation of Driest Month, bio_15 = Precipitation Seasonality, bio_16 = Precipitation of Wettest Quarter, bio_17 = Precipitation of Driest Quarter, bio_18 = Precipitation of Warmest Quarter, bio_19 = Precipitation of Coldest Quarter

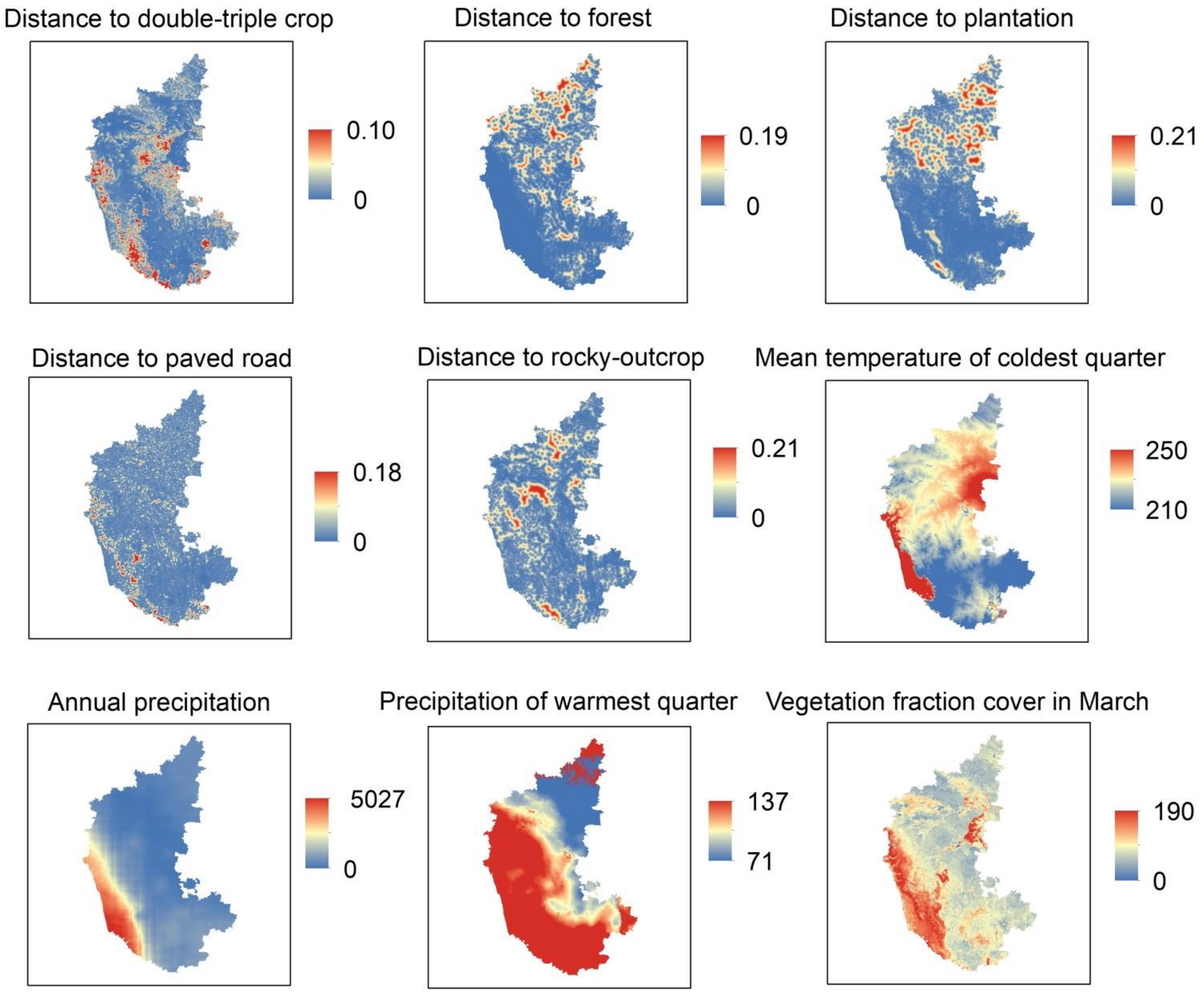

Figure S2. Variables selected to perform the model ensemble. The variables distance to double triple crop, distance to forest, distance to plantation, distance to rocky outcrops and distance to paved roads, are in WGS decimal degree where 0.1 = 10.06 kilometre. The variables, annual precipitation and precipitation of the warmest quarter are in millimetres.

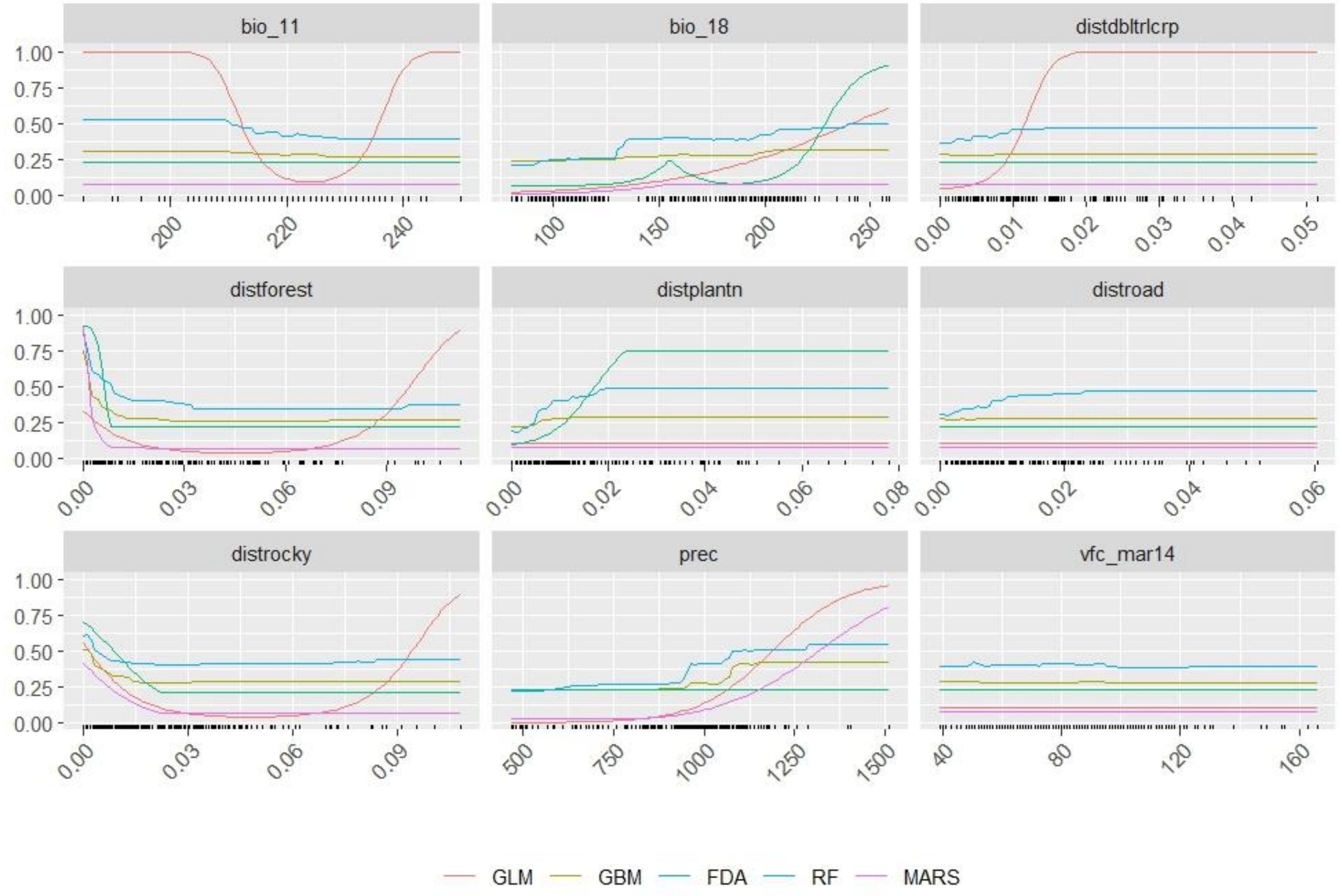

Figure S3. Variable response from the algorithms from Run 3 using evaluation strip Elith et al (2005). The variables distdbltrlcrp: distance to double triple crop, distforest : distance to forest, distplantn: distance to plantation, distrocky: distance to rocky outcrops and distroad: distance to paved roads, is in WGS decimal degree where 0.01 = 1.06 kilometre. The variables prec: annual precipitation and bio_18: precipitation of the warmest quarter is in millimetres. Vfc_mar14 is the vegetation fraction cover corresponding to March 2014.

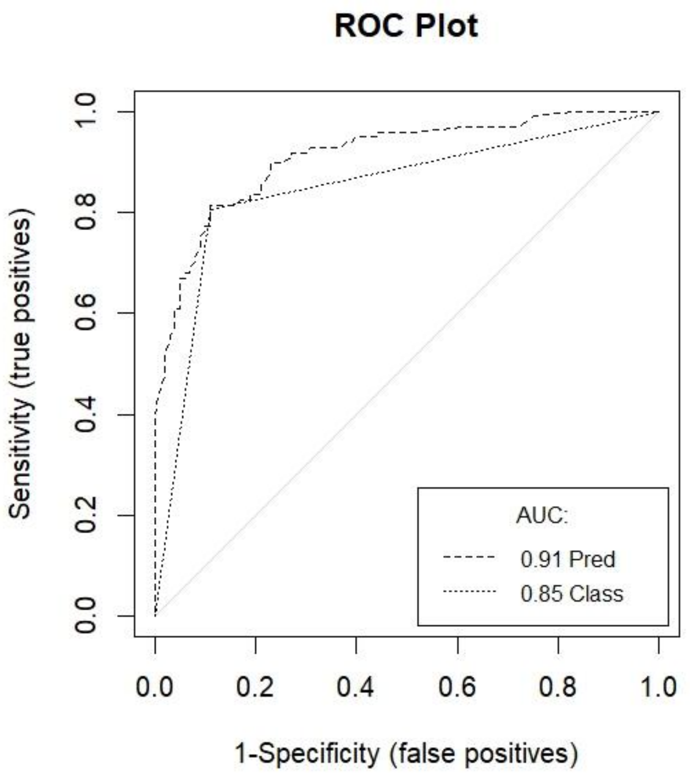

Figure S4. Relative Operating Characteristic (ROC) curve is shown for the model evaluation using an independent test set. Here AUC is the area under the curve.

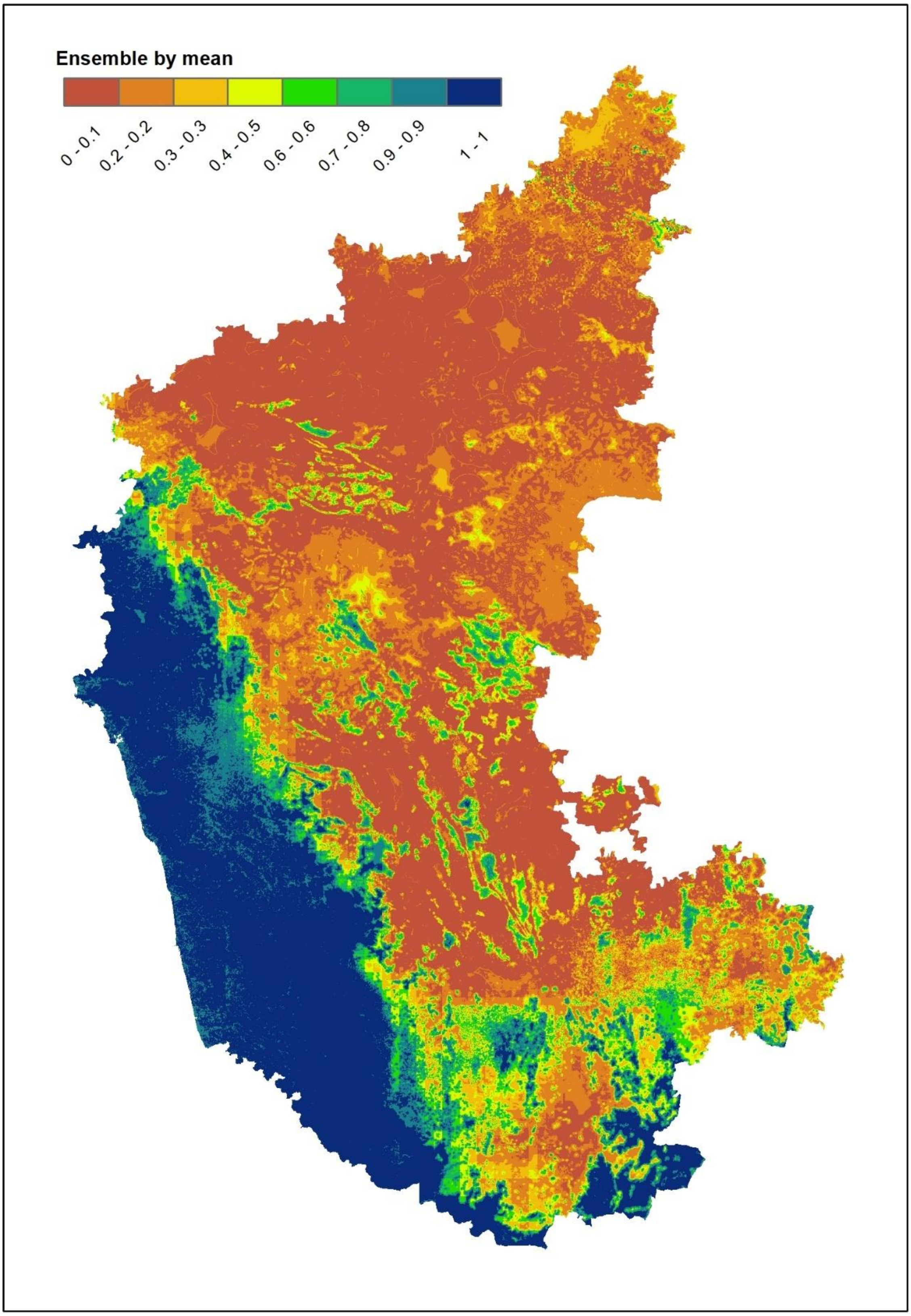

Figure S5: Predicted habitat selection for the Indian leopard (*Panthera pardus fusca*) using the consensus ensemble model projected in the study.

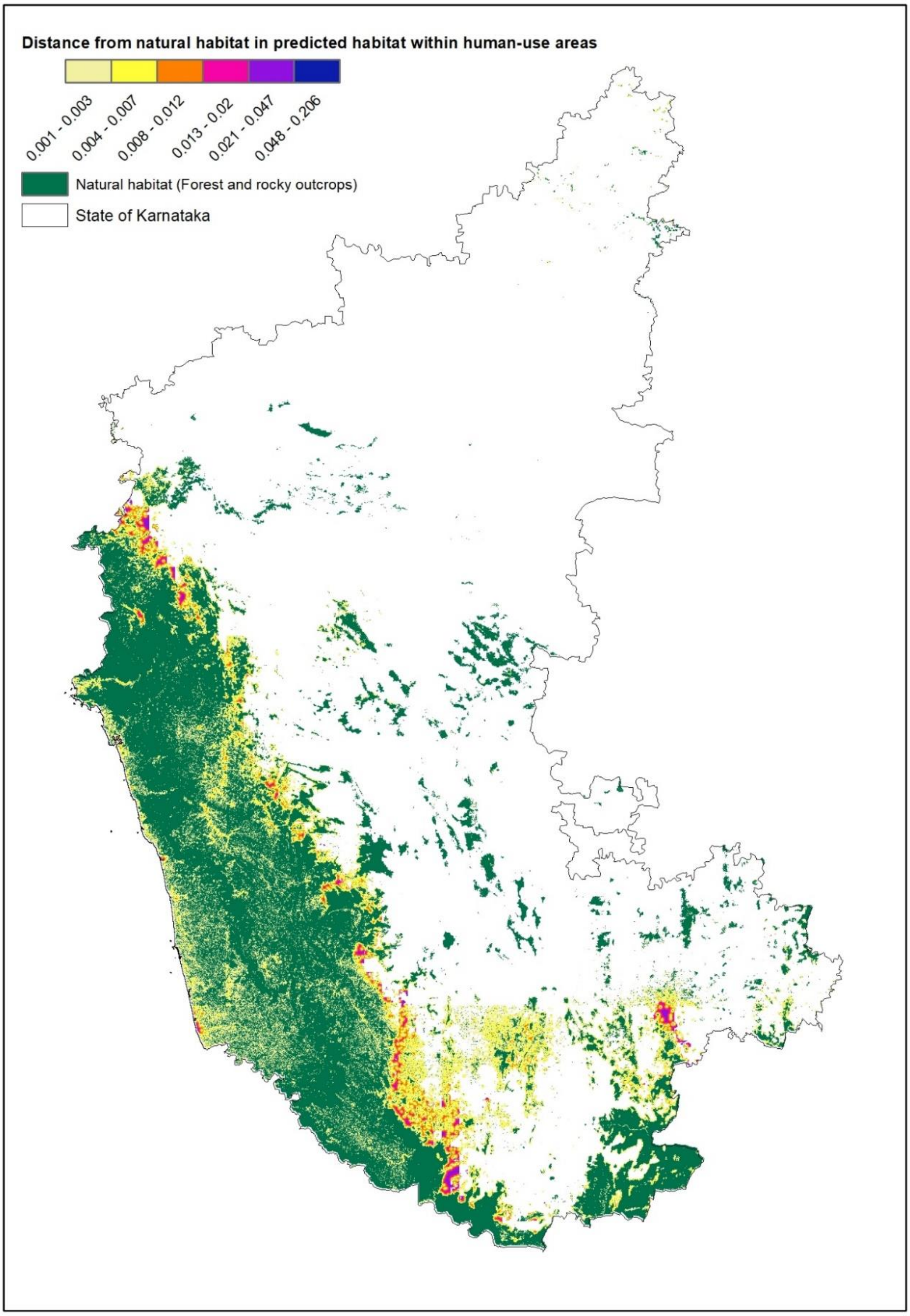

Figure S6: Euclidean distance mapping of the habitat selection predicted for leopards using the consensus ensemble model in human-use areas (built-up and croplands) to the nearest natural area. (Distance of 0.01 = 1.08 km)

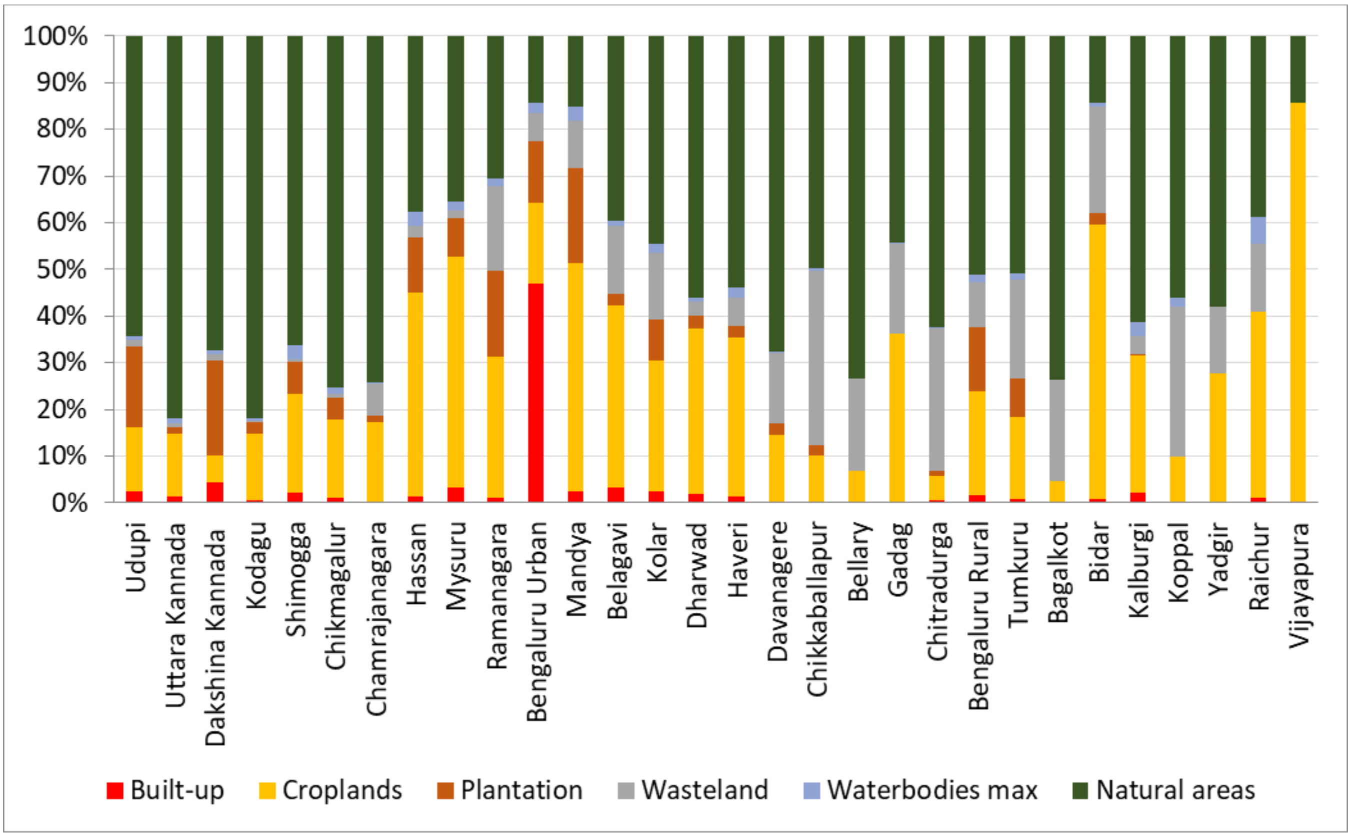

Figure S7: Percentage of land use category within the predicted habitat for Indian leopards in each of the districts in the state of Karnataka. Here, cropland is Rabi, Zaid, Kharif and current fallow croplands combined, natural areas are deciduous, evergreen, grassland and scrub forests combined. The districts are ordered on the largest habitat predicted to the lowest in the state.
